# Evaluation of Effects of Continuous Glucose Monitoring on Physical Activity Habits and Blood Lipid Levels in Persons With Type 1 Diabetes Managed With Multiple Daily Insulin Injections: An Analysis Based on the GOLD Randomized Trial (GOLD 8)

**DOI:** 10.1177/19322968221101916

**Published:** 2022-06-08

**Authors:** Thomas Nyström, Erik Schwarz, Sofia Dahlqvist, Magnus Wijkman, Magnus Ekelund, Helen Holmer, Jan Bolinder, Jarl Hellman, Henrik Imberg, Irl B. Hirsch, Marcus Lind

**Affiliations:** 1 Department of Clinical Science and Education, Södersjukhuset, Karolinska Institutet, Stockholm, Sweden; 2 Department of Internal Medicine, Faculty of Medicine and Health, Örebro University, Örebro, Sweden; 3 Department of Medicine, NU-Hospital Group, Uddevalla, Sweden; 4 Department of Molecular and Clinical Medicine, University of Gothenburg, Gothenburg, Sweden; 5 Department of Internal Medicine and Department of Medical and Health Sciences, Linköping University, Norrköping, Sweden; 6 Department of Clinical Sciences, Lund University, Lund, Sweden; 7 Department of Internal Medicine, Centralsjukhuset, Kristianstad, Sweden; 8 Department of Medicine, Karolinska University Hospital Huddinge, Karolinska Institutet, Stockholm, Sweden; 9 Department of Medical Sciences, Clinical Diabetes and Metabolism, Uppsala University, Uppsala, Sweden; 10 Statistiska Konsultgruppen, Gothenburg, Sweden; 11 Department of Mathematical Sciences, Chalmers University of Technology and University of Gothenburg, Gothenburg, Sweden; 12 School of Medicine, University of Washington, Seattle, WA, USA; 13 Department of Internal Medicine, Sahlgrenska University Hospital, Gothenburg, Sweden

**Keywords:** blood lipids, continuous glucose monitoring, hypoglycemia, low-grade inflammation, physical activity

## Abstract

**Background:**

People with type 1 diabetes generally view it easier to exercise when having continuous information of the glucose levels. We evaluated whether patients with type 1 diabetes managed with multiple daily insulin injections (MDI) exercised more after initiating continuous glucose monitoring (CGM) and whether the improved glycemic control and well-being associated with CGM translates into improved blood lipids and markers of inflammation.

**Method:**

The GOLD trial was a randomized cross-over trial over 16 months where patients used either CGM or capillary self-monitoring of blood glucose (SMBG) over six months, with a four-month wash-out period between the two treatment periods. We compared grade of physical activity, blood lipids, apolipoproteins, and high-sensitivity C-reactive protein (hsCRP) levels during CGM and SMBG.

**Results:**

There were 116 patients with information of physical activity estimated by the International Physical Activity Questionnaire (IPAQ) during both CGM and SMBG. No changes were found during CGM or SMBG, IPAQ scores 3305 versus 3878 (*P* = .16). In 136 participants with information of blood lipid levels with no change in lipid-lowering medication during the two treatment periods, HbA1c differed by 4.2 mmol/mol (NGSP 0.39%) between SMBG and CGM treatment (*P* < .001). No significant changes existed in low-density lipoprotein, high-density lipoprotein, triglycerides, total cholesterol, apolipoprotein A1, apolipoprotein B1, or hsCRP, during CGM and SMBG.

**Conclusion:**

Although many patients experience it easier to perform physical activity when monitoring glucose levels with CGM, it does not influence the amount of physical activity in persons with type 1 diabetes. Blood lipids, apolipoprotein, and hsCRP levels were similar during CGM and SMBG.

## Background

Type 1 diabetes is associated with increased risk of cardiovascular disease (CVD) and reduced life expectancy.^1-3^ People at high risk are those with long-term poor glycemic control and with renal complications, whereas persons with good glycemic control, without renal complications, and who are nonsmokers have a similar or close to similar CVD risk and life expectancy as persons without diabetes.^4-6^

Among other studies, the GOLD trial has shown that continuous glucose monitoring (CGM) improves glycemic control, and that glycemic variability decreases in persons with type 1 diabetes.^7,8^ Furthermore, treatment satisfaction and well-being were both improved during CGM in the GOLD trial, and patients also experienced a higher confidence regarding episodes of hypoglycemia.^7^ Besides the CVD preventive effect by lowering glucose levels, it is possible that the improved glucose levels and the reduced glycemic variability observed in the GOLD trial^7^ have an effect on other CVD risk factors such as blood lipids and apolipoprotein levels earlier described to be associated with the grade of glycemic control.^9^

In clinical practice many patients experience it easier to exercise during CGM compared with capillary self-monitoring of blood glucose (SMBG) due to having continuous information of the glucose levels before, during, and after exercise.^10^ It is therefore possible that variables such as physical activity may be influenced during CGM. The improved glycemic control and increased well-being and treatment satisfaction found by CGM^7^ may further be associated with less inflammation.^11^ Physical inactivity, due to lack of exercise, and low-grade systemic inflammation are important risk factors contributing to the pathogenesis of CVD.^12,13^

In the current post hoc analysis of the GOLD trial,^7^ we evaluated whether CGM compared with SMBG influences the grade of physical activity, blood lipid levels, apolipoprotein levels, and high-sensitivity C-reactive protein (hsCRP) levels. We also used the current cohort to evaluate whether an overall association exists between changes in glycemic control and glycemic variability with blood lipids, apolipoproteins, or hsCRP irrespective of glucose monitoring method.

## Methods

The GOLD trial was approved by the ethics committee at the University of Gothenburg, Gothenburg, Sweden (diary number 857-13). The design and results of key endpoints including effects on glycemic control, hypoglycemia, treatment satisfaction, hypoglycemia confidence, diabetes-related distress, and well-being have earlier been presented.^5,7,8^ In brief, all participants gave verbal and written informed consent. The study was an investigator-initiated randomized, open-label, clinical trial with a cross-over design conducted at 15 sites in Sweden. Patients monitored their glucose levels by CGM over six months and by SMBG using capillary finger-stick testing over six months, with a four-month wash-out period between the two treatment periods. During SMBG, masked CGM was performed during two of the four last weeks of the six months follow-up period to have comparative CGM data during the two treatment periods.

Individuals 18 years or older with hemoglobin A1c (HbA1c) ≥58 mmol/mol (NGSP 7.5%) managed with multiple daily insulin injections (MDI) were included. Patients were required to have a fasting C-peptide level <0.3 nmol/L (0.91 ng/mL) and diabetes duration of more than one year. Patients treated with insulin pumps were excluded. All laboratory tests were analyzed at a central laboratory (Research Centre for Laboratory Medicine, Karolinska University Hospital, Stockholm, Sweden).

### Physical Activity

Patients were not asked, or particularly encouraged to exercise during the study. Grade of physical activity was recorded by the International Physical Activity Questionnaire (IPAQ).^14^ IPAQ consists of four questions of various levels of physical activity during the last seven days. Participants recorded physical activity in the IPAQ questionnaire during the run-in period before randomization, at the end of the first treatment period (week 26), at the start of the second treatment period (week 43), and at the end of the second treatment period (week 69).

MET values (multiples of the resting metabolic rate) and formula for computation of MET-minutes were as follows:

• Inactive MET-minutes/week = 3.3 × walking minutes × walking days.
• Minimal active MET-minutes/week = 4.0 × moderate-intensity activity minutes × moderate days.
• Vigorous active MET-minutes/week = 8.0 × vigorous-intensity activity minutes × vigorous-intensity days.

A combined total physical activity MET-min/week was computed as the sum of inactive + minimal active + vigorous active MET-min/week scores, that is, IPAQ total score.^14^

### Continuous Glucose Monitoring and Self-Monitoring of Blood Glucose

Patients were randomized and unblinded to either CGM (DexCom G4 PLATINUM stand-alone system) or conventional therapy (SMBG). All patients received basic instruction on insulin dosing and bolus correction, food choices, and the potential effect of physical activity on glucose control. This information was provided at the same level as in clinical practice for patients with type 1 diabetes and to guarantee that patients have basic skills for dosing insulin, and what food choices and physical activity may affect glucose control.^7^ A graph was displayed for patients showing the proportion of insulin at time of injection (100%) and the proportion of insulin remaining to give effect at various time points after injection. The patients received general guidelines for interpreting glucose levels and trends obtained by CGM.^7^

### Blood Lipid Levels, Apolipoproteins, hsCRP, Creatinine, and HbA1c

At the start and end of each treatment period (CGM and SMBG), blood lipid levels, that is, low-density lipoprotein (LDL), high-density lipoprotein (HDL), triglycerides and total cholesterol and apolipoproteins, that is, apolipoprotein A1 (ApoA1) and apolipoprotein B1 (ApoB1) were measured in a fasting condition. hsCRP, creatinine, and HbA1c were also measured at the same time points.

### Blood Pressure and Weight

Systolic and diastolic blood pressure were measured with the patient in a seated position after 5 to 10 minutes rest at the start and end of each treatment period. Weight was measured in a standing position, with light clothing without shoes, on a calibrated measuring scale.

### Procedures

In the current exploratory analyses (GOLD 8), we analyzed the following variables at the end of the six months of CGM and SMBG treatment periods: physical activity by the IPAQ questionnaire, weight, blood pressure, total cholesterol, LDL-cholesterol, HDL-cholesterol, triglycerides, ApoA1 and ApoB1, hsCRP, creatinine, and HbA1c. Only patients with stable, or no lipid-lowering/antihypertensive, medication, were included in the respective analyses, that is, Full Analysis Set (FAS).

Changes in HbA1c, time in range (TIR) and measures for glycemic variability,^15^ in the form of standard deviation (SD), coefficient of variation (CV), and mean amplitude of glycemic excursions (MAGE), were analyzed during CGM and SMBG for patients with information of blood lipid and apolipoprotein levels. Body weight, total insulin dose, and insulin dose/kg were also analyzed during CGM and SMBG in the same patient cohort. Subgroup analyses were performed for subjects with HbA1c reductions ≥5 mmol/mol (NGSP ≈ 0.5%), ≥7 mmol/mol (NGSP ≈ 0.7%), or ≥10 mmol/mol (NGSP ≈ 1%) during CGM compared with SMBG. Similar analyses were also performed in a subgroup of patients with weight reduction, CGM SD reduction, CGM CV reduction, MAGE reduction, and TIR increase above the 50th and 75th percentiles.

To evaluate whether an overall association existed between HbA1c level and the studied variables, the correlation coefficient between change in HbA1c and change in blood lipids, apolipoprotein, and hsCRP levels during CGM and SMBG was calculated. Similar analyses were also performed for change in glycemic variability (SD, CV, and MAGE), time in hypoglycemia (<3.0 and <3.9 mmol/L), and TIR related to change in blood lipids, apolipoproteins, and hsCRP levels.

### Statistics

All continuous variables were described by mean, SD, median, and range. Categorical variables were described as numbers (n) and percentage (%). The main analyses were performed using general linear models with sequence (CGM-SMBG or SMBG-CGM), patient (nested within sequence), treatment period (1 or 2), and treatment (CGM or SMBG) as explanatory variables for normally distributed variables, and using nonparametric permutation test for the difference in means for non-normally distributed variables. Subjects with missing data on one of the treatment periods were excluded from the analyses. Correlation analyses were performed using Spearman nonparametric rank correlation coefficient. All tests were two-tailed and conducted at .05 significance level. All analyses were performed using SAS software version 9.4 (SAS Institute Inc., Cary, North Carolina).

## Results

Patient characteristics and the evaluated variables at baseline before randomization are presented in Table 1. Blood lipids and blood pressure levels were similar when excluding patients with changing lipid-lowering medication during follow-up (n = 6; Supplemental material, Table S1), or patients with changing antihypertensive treatment during follow-up (n = 19; Supplemental material, Table S2).

**Table 1.** Demographics and Patient Characteristics (FAS Population).

Variable	Sequence 1: CGM-SMBG (n = 69)	Sequence 2: SMBG-CGM (n = 73)	*P* value
**Demographics and baseline characteristics**			
Age (years)	46.7 (13.0) 47 (20-77) n = 69	42.6 (12.2) 43 (19-77) n = 73	.06
Female sex	32 (46.4%)	30 (41.1%)	.64
Caucasian race	69 (100.0%)	72 (98.6%)	1.00
Non-Hispanic/Latino	69 (100.0%)	73 (100.0%)	1.00
Diabetes duration at randomization (years)	23.4 (11.9) 23.1 (3-56.6) n = 69	21.0 (11.7) 18.8 (1.4-44.2) n = 73	.23
Smoking			
Current	7 (10.1%)	10 (13.7%)	
Previous	17 (24.6%)	15 (20.5%)	
Never	45 (65.2%)	48 (65.8%)	.80
**Medical history**			
Previous laser photocoagulation of the retina	14 (20.3%)	14 (19.2%)	1.00
Previous myocardial infarction	3 (4.3%)	0 (0%)	.22
Previous stroke	1 (1.4%)	1 (1.4%)	1.00
Previous bypass-graft	1 (1.4%)	0 (0%)	.97
Previous percutaneous coronary intervention	2 (2.9%)	0 (0%)	.47
Previous amputation	0 (0%)	1 (1.4%)	1.00
Previous diabetic foot or leg ulcer	1 (1.4%)	5 (6.8%)	.24
Current diabetic foot or leg ulcer	0 (0%)	3 (4.1%)	.27
Self-estimated number of hypoglycemias per week during the last two months	1.90 (1.48) 1.75 (0-7) n = 66	2.36 (2.23) 2 (0-12) n = 68	.18
Number of severe hypoglycemias past year	0.101 (0.425) 0 (0-3) n = 69	0.042 (0.262) 0 (0-2) n = 72	.48
Number of severe hypoglycemias past 5 years	0.884 (3.042) 0 (0-20) n = 69	0.319 (0.709) 0 (0-4) n = 72	.17
Lipid lowering medication at randomization	33 (47.8%)	34 (46.6%)	1.00
Number of blood pressure lowering medications at randomization			
0	39 (56.5%)	50 (68.5%)	
1	17 (24.6%)	16 (21.9%)	
2	7 (10.1%)	2 (2.7%)	
≥3	6 (8.7%)	5 (6.8%)	.13
**HbA1c at Run-in and Randomization**			
HbA1c at Run-in (mmol/mol)	71.7 (8.8) 71 (58-103) n = 69	71.6 (9.5) 69 (58-104) n = 73	.97
HbA1c at Run-in (%)	8.71 (0.81) 8.65 (7.46-11.58) n = 69	8.70 (0.87) 8.47 (7.46-11.67) n = 73	.97
HbA1c at Visit 4 Randomization (mmol/mol)	69.2 (9.5) 69 (50-103) n = 69	68.8 (9.4) 67 (55-102) n = 72	.79
HbA1c at Visit 4 Randomization (%)	8.49 (0.87) 8.47 (6.73-11.58) n = 69	8.45 (0.86) 8.28 (7.19-11.49) n = 72	.79
**Laboratory data at Randomization**			
Total cholesterol (mmol/L)	4.59 (0.99) 4.5 (2.8-7.6) n = 69	4.42 (0.93) 4.4 (2.4-6.7) n = 72	.30
Low-density lipoprotein (mmol/L)	2.45 (0.74) 2.4 (1.3-5.6) n = 68	2.50 (0.79) 2.4 (1-4.6) n = 72	.73
High-density lipoprotein (mmol/L)	1.71 (0.59) 1.5 (0.9-3.5) n = 69	1.50 (0.41) 1.4 (0.8-3.3) n = 72	.018
Triglycerides (mmol/L)	0.972 (0.744) 0.76 (0.25-4.4) n = 69	0.951 (0.546) 0.815 (0.36-3.9) n = 72	.85
Apolipoprotein A1 (g/L)	1.72 (0.36) 1.66 (1.08-2.8) n = 69	1.56 (0.28) 1.53 (0.96-2.64) n = 72	.004
Apolipoprotein B1 (g/L)	0.865 (0.184) 0.85 (0.47-1.47) n = 69	0.875 (0.216) 0.84 (0.5-1.63) n = 72	.76
High-sensitivity C-reactive protein (mg/L)	1.99 (2.62) 1.3 (0.2-17.9) n = 69	3.48 (7.13) 1.55 (0.2-54.2) n = 72	.09
Creatinine (µmol/L)	72.6 (14.4) 71 (41-109) n = 69	70.1 (13.3) 68.5 (37-112) n = 72	.30
**Blood pressure at Randomization**			
Systolic blood pressure (mmHg)	125.4 (12.0) 126 (95-164) n = 69	126.1 (19.6) 125 (80-200) n = 72	.81
Diastolic blood pressure (mmHg)	73.4 (9.9) 74 (52-92) n = 69	74.3 (9.6) 72.5 (60-100) n = 72	.59
**Physical activity at Run-in**			
IPAQ Total Score	3628 (4524) 1760 (80-23 730) n = 61	3158 (3389) 1949 (0-13 880) n = 68	.51
IPAQ Total Score, categorical			
Inactive	40 (58.8%)	46 (63.0%)	
Minimally Active	7 (10.3%)	4 (5.5%)	
Vigorous Active	21 (30.9%)	23 (31.5%)	.82

Data are presented as mean (SD), median (range), and numbers for continuous variables, as and number (%) for categorical variables. For comparison between groups, Fishers exact test (2 × lowest one-sided *P* value) was used for binary variables, Mantel-Haenszel chi-square test for ordered categorical variables, and nonparametric permutation test for the difference in means for continuous variables.

Abbreviations: CGM, continuous glucose monitoring; FAS, Full Analysis Set; HbA1c, hemoglobin A1c; IPAQ, International Physical Activity Questionnaire; SD, standard deviation; SMBG, self-monitoring of blood glucose.

After exclusion of patients with changes in lipid-lowering medications, the mean difference in HbA1c between treatments was 4.2 mmol/mol (NGSP 0.39 %), *P* < .001, in favor of CGM use. Results for the main analyses regarding grade of physical activity, blood lipid levels, ApoA1/ApoB1 levels, and hsCRP levels during CGM and SMBG treatments are shown in Table 2. There were no differences in grade of physical activity during CGM compared with SMBG, IPAQ scores 3305 versus 3878 (*P* = .16; Supplemental material, Figure S1). There were no differences with respect to insulin doses, or in any measure of blood lipid levels, or ApoA1/ApoB1 levels between treatments (Table 2). hsCRP levels, weight, blood pressure, and creatinine levels were also similar during both treatment periods (Table 2).

**Table 2.** Changes Between Treatments (CGM vs SMBG) With Respect to Physical Activity Habits (IPAQ Total Score), High-Sensitivity C-Reactive Protein Levels, Blood Lipids, Apolipoproteins, HbA1c, Weight, Blood Pressure, and Creatinine.

	CGM (DexCom G4)	Conventional therapy (SMBG)	Difference CGM-SMBG
***FAS population***			
IPAQ Total Score	3305 (3338) 2246 (50-17 160) n = 116	3878 (4701) 1875 (120-24 612) n = 116	−573 (4277) 46 (−22 376-12 318) *P* = .16
High-sensitivity C-reactive protein (mg/L)	3.70 (7.75) 1.4 (0.2-51.5) n = 129	3.15 (8.19) 1.2 (0.2-80.6) n = 129	0.54 (10.69) 0.0 (−77.8-50.8) *P* = .59
***FAS population excluding changed lipid-lowering medication***			
Total cholesterol (mmol/L)	4.52 (0.87) 4.5 (2.6-7.2) n = 125	4.43 (0.85) 4.3 (2.5-7.7) n = 125	0.07 (−0.07-0.21) *P* = .30
Low-density lipoprotein (mmol/L)	2.50 (0.72) 2.5 (1.0-5.0) n = 123	2.43 (0.74) 2.4 (0.7-5.1) n = 123	0.06 (−0.05-0.17) *P* = .29
High-density lipoprotein (mmol/L)	1.59 (0.48) 1.5 (0.8-3.1) n = 125	1.58 (0.44) 1.5 (0.8-2.8) n = 125	0.01 (0.31) 0.0 (−0.7-1.4) *P* = .82
Triglycerides (mmol/L)	0.96 (0.55) 0.8 (0.3-4.1) n = 125	0.98 (0.66) 0.8 (0.3-5.5) n = 125	−0.03 (0.58) 0.0 (−4.1-2.4) *P* = .65
Apolipoprotein A1 (g/L)	1.59 (0.33) 1.5 (1.0-3.0) n = 125	1.60 (0.28) 1.6 (1.0-2.4) n = 125	−0.00 (−0.04-0.04) *P* = .89
Apolipoprotein B1 (g/L)	0.84 (0.19) 0.9 (0.5-1.6) n = 125	0.83 (0.20) 0.8 (0.4-1.5) n = 125	0.02 (−0.01-0.05) *P* = .19
High-sensitivity C-reactive protein (mg/L)	3.61 (7.69) 1.4 (0.2-51.5) n = 125	3.15 (8.30) 1.2 (0.2-80.6) n = 125	0.46 (10.75) 0.0 (−77.8-50.8) *P* = .66
HbA1c (mmol/mol)	62.80 (8.87) 62.0 (41.0-84.0) n = 125	67.04 (9.06) 66.0 (49.0-96.0) n = 125	−4.24 (−5.78-2.69) *P* < .0001
HbA1c (%)	7.90 (0.81) 7.8 (5.9-9.8) n = 125	8.29 (0.83) 8.2 (6.6-10.9) n = 125	−0.39 (−0.53-0.25) *P* < .0001
Weight (kg)	83.82 (16.45) 82.6 (51.0-133.2) n = 125	83.30 (16.31) 82.9 (51.9-129.2) n = 125	0.49 (−0.01-0.99) *P* = .052
Total daily insulin dose (units)	56.64 (25.81) 51.0 (23.0-148.0) n = 125	57.46 (25.98) 52.0 (23.0-170.0) n = 125	−0.92 (−2.57-0.72) *P* = .27
Total daily insulin dose per kg (units/kg)	0.66 (0.22) 0.6 (0.3-1.4) n = 125	0.68 (0.23) 0.6 (0.4-1.7) n = 125	−0.02 (−0.04-0.00) *P* = .08
***FAS population excluding changed blood pressure–lowering medication***			
Systolic blood pressure (mmHg)	122.0 (12.5) 120 (91-161) n = 119	122.1 (13.0) 121 (90-160) n = 119	−0.13 (−2.17-1.91) *P* = .90
Diastolic blood pressure (mmHg)	73.8 (8.9) 75 (54-92) n = 119	72.3 (8.8) 72 (52-92) n = 119	1.42 (−0.18-3.02) *P* = .082
Creatinine (µmol/L)	75.56 (14.17) 76.0 (46.0-116.0) n = 123	74.76 (16.59) 74.0 (37.0-128.0) n = 123	0.62 (−0.93-2.16) *P* = .43

Data during CGM or SMBG are presented as mean (SD), median (range), and number of observations. Differences between treatments (CGM-SMBG) are presented as mean difference with 95% confidence interval for normally distributed variables, and as mean (SD) and median (range) for non-normally distributed variables. Analyses of normally distributed variables were performed using general linear models with sequence (CGM-SMBG or SMBG-CGM), patient (nested within sequence), period (1 or 2), and treatment as explanatory variables. Analyses of non-normally distributed variables were performed using nonparametric permutation test for the difference in means. Subjects with missing data on one of the treatment periods were excluded from the analyses.

Abbreviations: CGM, continuous glucose monitoring; FAS, Full Analysis Set; HbA1c, hemoglobin A1c; IPAQ, International Physical Activity Questionnaire; SD, standard deviation; SMBG, self-monitoring of blood glucose.

### Subgroup Analyses in Patients With Larger HbA1c and Weight Reductions

There were no significant differences in blood lipids, apolipoproteins, or in hsCRP levels between treatments when evaluated in subgroups of patients with HbA1c reductions ≥5 mmol/mol (NGSP ≈ 0.5%), ≥7 (NGSP ≈ 0.7%), or ≥10 mmol/mol (NGSP ≈ 1%) during CGM compared with SMBG treatment (Supplemental material, Tables S3-S5).

As weight reduction may change lipid levels and low-grade inflammation, we also evaluated treatment effects in subgroups with weight change ≤0.7 kg (50th percentile) or weight reduction ≥1.0 kg (75th percentile) during CGM compared with SMBG treatments. There were no significant differences in blood lipids, apolipoproteins, and hsCRP levels between treatments in any of these subgroups (Supplemental material, Tables S6-S7).

### Subgroup Analyses in Patients With Larger Reductions in Glucose Variability (SD, CV, and MAGE) or Larger Changes in Time in Hypoglycemia and TIR

High glucose variability may influence lipid metabolism and inflammatory mechanisms. We therefore investigated these variables in subgroups of patients with larger reductions in glucose variability (SD, CV, and MAGE) or larger increase in TIR during CGM compared with SMBG. Subgroup analyses did not reveal any differences in blood lipids, apolipoproteins, or hsCRP levels between treatments in any of the subgroups (Supplemental material, Tables S8-S15).

### Overall Association Between Changes in Glycemic Control, Glucose Variability, Weight, Lipids, Apolipoproteins, hsCRP, and IPAQ Score

Finally, we evaluated the overall association between changes in glycemic control (HbA1c), glucose variability (SD, CV, and MAGE), weight, proportion of time in hypoglycemia (<3.0 and <3.9 mmol/L), and TIR with changes in blood lipids, apolipoproteins, hsCRP levels, and IPAQ score during CGM versus SMBG (Table 3). There was a weak positive association between changes in HbA1c and changes in triglyceride levels (*r* = .19, *P* = .034) and a borderline negative association between changes in TIR and total cholesterol (*r* = −.18, *P* = .053) and LDL-cholesterol (*r* = −.18, *P* = .053), respectively.

**Table 3.** Spearman Correlation Coefficients Between Changes (CGM vs SMBG) in Glycemic Control (HbA1c, Time in Hypoglycemia, and Time in Range), Glucose Variability (SD, CV, and MAGE), Weight, Lipids, Apolipoproteins, hsCRP, and IPAQ Score (FAS Population Excluding Subjects With Changes in Lipid-Lowering Medication).

	HbA1c	CGM SD	CGM CV	MAGE	Weight	Time in hypoglycemia <3.0 mmol/L	Time in hypoglycemia <3.9 mmol/L	Time in range 3.9-10 mmol/L
Total cholesterol (mmol/L)	*r* = .16 *P* = .07	*r* = −.02 *P* = .84	*r* = −.06 *P* = .54	*r* = −.07 *P* = .44	*r* = −.01 *P* = .94	*r* = .06 *P* = .53	*r* = −.01 *P* = .96	*r* = −.18 *P* = .053
LDL (mmol/L)	*r* = .17 *P* = .06	*r* = −.02 *P* = .80	*r* = −.12 *P* = .21	*r* = −.09 *P* = .35	*r* = .02 *P* = .80	*r* = .01 *P* = .94	*r* = −.04 *P* = .71	*r* = −.18 *P* = .053
HDL (mmol/L)	*r* = −.01 *P* = .92	*r* = −.02 *P* = .86	*r* = .09 *P* = .33	*r* = −.08 *P* = .40	*r* = −.10 *P* = .29	*r* = .07 *P* = .45	*r* = .03 *P* = .78	*r* = −.04 *P* = .68
Triglycerides (mmol/L)	*r* = .19 *P* = .03	*r* = −.002 *P* = .98	*r* = −.16 *P* = .09	*r* = −.01 *P* = .96	*r* = −.04 *P* = .66	*r* = −.02 *P* = .81	*r* = −.06 *P* = .52	*r* = −.09 *P* = .36
Apolipoprotein A1 (g/L)	*r* = −.04 *P* = .65	*r* = −.04 *P* = .63	*r* = .11 *P* = .24	*r* = −.05 *P* = .60	*r* = −.12 *P* = .19	*r* = .03 *P* = .79	*r* = .01 *P* = .93	*r* = .06 *P* = .51
Apolipoprotein B1 (g/L)	*r* = .10 *P* = .27	*r* = .09 *P* = .35	*r* = −.03 *P* = .71	*r* = .12 *P* = .21	*r* = −.12 *P* = .19	*r* = .02 *P* = .84	*r* = −.04 *P* = .72	*r* = −.14 *P* = .13
hsCRP (mg/L)	*r* = .04 *P* = .69	*r* = .00 *P* = .99	*r* = −.06 *P* = .53	*r* = .04 *P* = .67	*r* = .00 *P* = .98	*r* = −.14 *P* = .16	*r* = −.12 *P* = .20	*r* = −.03 *P* = .76
IPAQ total score	*r* = .03 *P* = .77	*r* = .07 *P* = .52	*r* = .04 *P* = .68	*r* = .05 *P* = .64	*r* = .18 *P* = .07	*r* = .15 *P* = .15	*r* = .08 *P* = .44	*r* = .10 *P* = .35

*r* is the Spearman correlation coefficient.

Abbreviations: CGM, continuous glucose monitoring; CV, coefficient of variation; HbA1c, hemoglobin A1c; HDL, high-density lipoprotein; hsCRP, high-sensitivity C-reactive protein; IPAQ, International Physical Activity Questionnaire; LDL, low-density lipoprotein; MAGE, mean amplitude of glycemic excursions; MET, multiples of the resting metabolic rate; SMBG, self-monitoring of blood glucose; SD, standard deviation.

## Discussion

In this first analysis of the grade of physical activity, blood lipid and apolipoprotein levels from a randomized trial of CGM versus SMBG in persons with type 1 diabetes, we found no changes in blood lipid and apolipoprotein levels, or grade of physical activity. Low-grade inflammation, measured as hsCRP levels, was also similar during both treatment phases. For patients with larger changes in glycemic control, glycemic variation, or weight between treatment periods, no changes in blood lipids, apolipoproteins, or hsCRP levels were found between treatments. However, there was an overall weak positive correlation observed between change in HbA1c and change in triglyceride levels during the study.

It is well known that there is an association between glucose metabolism and lipid metabolism in human.^16^ One important clinical example is diabetic dyslipidemia in which hyperglycemia, typically in people with type 2 diabetes with insulin resistance, has increased triglyceride and LDL-cholesterol levels, concomitant with decreased HDL-cholesterol levels.^17^ Diabetic dyslipidemia is also observed in overweight people with type 1 diabetes^18^ and in persons with poor glycemic control.^19,20^ As the use of CGM resulted in lower HbA1c level in the GOLD trial,^7^ this current post hoc GOLD 8 study aimed to investigate whether there was any association between glycemic control and blood lipid and apolipoprotein levels. If such an effect would exist, CGM would have additional effects not only on the glucose control but also on the blood lipid profile.

Even though the use of CGM significantly lowered HbA1c, there were no changes in blood lipids or apolipoprotein levels between groups, nor was there any association between higher changes in HbA1c reduction and blood lipids or apolipoprotein levels, in patients during the use of CGM compared with SMBG.

There might be several reasons for the lack of changes in blood lipids during CGM treatment. A mean change in HbA1c of 4.2 mmol/mol (NGSP 0.39 %) may simply not be enough to interact on blood lipid and apolipoprotein levels. Notwithstanding this a HbA1c reduction of approximately 4 to 5 mmol/mol is probably enough to reduce long-term complications in people with type 1 diabetes.^21^ However, after categorizing patients in more powerful action on HbA1c reduction during the CGM treatment, there was still no significant changes, or associations regarding blood lipid, or apolipoprotein levels between treatment groups. Furthermore, patients in the current study were well-controlled in blood lipid profile, and it is likely that glycemic reduction might have had a better effect on patients with uncontrolled blood lipid profile.^22^ Recent studies have suggested that not only increased HbA1c but increased glycemic variation may contribute to excess cardiovascular risk in people with diabetes.^23,24^ In the GOLD trial, the use of CGM did not only reduce HbA1c, but glycemic variation.^7^ Despite this, glycemic variation did not associate with the changes (in any of the categorized subgroups), of blood lipid and apolipoprotein levels, during the treatments.

Recently, low-grade inflammation has been suggested as one potential explanation for the increased cardiovascular risk observed in people with diabetes.^25^ In many studies, hsCRP has been used as a marker of chronic low-grade inflammation, and an association between high levels of hsCRP and cardiovascular outcomes in people with diabetes has been shown.^26^ In people with type 1 diabetes, nephropathy also contributes to low-grade inflammation.^27^ Not only hyperglycemia induces chronic inflammation, but intermittent hypoglycemia which may induce oxidative stress and endothelial dysfunction contributing to the excess risk of cardiovascular complications observed in people with diabetes.^28^ In the current post hoc analysis of data from the GOLD trial, there was no interaction between glycemic control or glycemic variation and hsCRP levels between treatments, nor did changes of creatinine associate with hsCRP levels, suggesting that despite improvement in glycemic control and glucose variability, the use of CGM has no effect on low-grade inflammation.

One lowest common denominator that may affect risk factors such as glycemic control, blood lipids, chronic inflammation, and weight gain is physical activity.^29^ CGM has been used frequently in exercise studies in people with type 1 diabetes.^10^ Some people with type 1 diabetes are insecure of insulin dosing and afraid of hypoglycemia close to physical activity and therefore may refrain from heavy exercise. In the GOLD trial, there were less time spent in hypoglycemia during CGM and numerically more severe hypoglycemic episodes during SMBG compared with during CGM (5 vs 1), and the hypoglycemia confidence questionnaire scale showed less hypoglycemia fear during CGM.^7^ Also, overall well-being, estimated with the World Health Organization (WHO)-5 questionnaire scale, was improved in the patients during CGM use. Despite improvement in these scales, there were no changes in the grade of physical activity habits, estimated with the IPAQ scale, between treatments. The reason for the lack of improvement of physical activity during CGM use is not clear. Even though patients may experience that exercise is easier by using CGM,^10^ and that a recent observational study demonstrated that the use of CGM associates with increase in exercise,^30^ patients in the current study did not improve the grade of physical activity habits. Although any reduction of hypoglycemia is of great importance for the patients, this may suggest that other factors, for example, motivation, rather than reducing fear of hypoglycemia, are more essential factors for increasing physical activity for persons with type 1 diabetes.

In the current post hoc analysis of the GOLD study, patients randomized to CGM were approximately gaining a half kilogram in weight (borderline significant). This was observed without a concomitant increase in insulin dosing, and the change in weight was not associated with the cardiovascular risk markers of interest. The minimal weight gain in the current study is less than in other longer interventional studies^21^ and may not raise any concerns. On the contrary, there are weight loss studies in which interventions have great impact on cardiovascular risk markers.^31^ For example, bariatric surgery in people with severe obesity is associated with decreased overall mortality^32^ and prevention of type 2 diabetes.^33^ Today, it is not uncommon that people with type 1 diabetes are overweight and due to that insulin-resistant.^18^ This may guide preventive measures by improving risk assessment in which weight is one important risk factor.^34^

### Strengths

To the best of our knowledge, this is the first evaluation based on a randomized controlled trial comparing the use of CGM and SMBG on physical activity habits and changes in blood lipid levels and markers of chronic inflammation. For that reason, we had full control over critical medications, that is, statins and blood pressure medications that may interfere with our outcomes. Another strength is that masked CGM was performed during the SMBG period to characterize glucose levels during SMBG monitoring.

### Limitations

This study had several limitations. Physical activity was self-reported without any objective measurements, for example, accelerometer. Also, the low number of physically active subjects in combination with no encouragement to be physically active in the study design might have contributed to the lack of increased exercise. Although blood lipids were measured at the start and end of each treatment period at predefined time points, repeated measurements were not performed during the treatment periods. Furthermore, although the majority of patients (n = 125) had information of blood lipids in both treatment phases, it should be noted that 11% of the original cohort^7^ lacked pairwise measurements. Generally, in parallel group studies, this can to some extent lead to an imbalance between groups. However, in the current study, patients served as their own controls, and thus no such problem existed. In contrast, we cannot rule out that the small sample size along with a potentially large relative variability in results may have led to the lack of significance. Finally, this was a post hoc study from the main GOLD trial^7^ and therefore the results must be seen as hypothesis generating.

## Conclusions

In conclusion, although many persons with type 1 diabetes experience it easier to perform physical activity when monitoring glucose control with CGM, it did not influence the magnitude of physical activity. Risk markers of CVD such as blood lipids, apolipoprotein, and hsCRP levels were similar during CGM and SMBG.

## Supplemental Material

sj-docx-1-dst-10.1177_19322968221101916 – Supplemental material for Evaluation of Effects of Continuous Glucose Monitoring on Physical Activity Habits and Blood Lipid Levels in Persons With Type 1 Diabetes Managed With MDI: An Analysis Based on the GOLD Randomized Trial (GOLD 8) Supplemental material, sj-docx-1-dst-10.1177_19322968221101916 for Evaluation of Effects of Continuous Glucose Monitoring on Physical Activity Habits and Blood Lipid Levels in Persons With Type 1 Diabetes Managed With MDI: An Analysis Based on the GOLD Randomized Trial (GOLD 8) by Thomas Nyström, Erik Schwarz, Sofia Dahlqvist, Magnus Wijkman, Magnus Ekelund, Helen Holmer, Jan Bolinder, Jarl Hellman, Henrik Imberg, Irl B. Hirsch and Marcus Lind in Journal of Diabetes Science and Technology
