# Supplementary material for: Evaluation of Effects of Continuous Glucose Monitoring on Physical Activity Habits and Blood Lipid Levels in Persons With Type 1 Diabetes Managed With Multiple Daily Insulin Injections: An Analysis Based on the GOLD Randomized Trial (GOLD 8)
Source: J Diabetes Sci Technol. 2022 Jun 8;18(1):89–98. doi: 10.1177/19322968221101916 (PMC10899843; doi:10.1177/19322968221101916)

**Supplementary Material**

Evaluation of effects of continuous glucose monitoring on physical activity habits and blood lipid levels in persons with type 1 diabetes managed with MDI: An analysis based on the GOLD randomized trial (GOLD 8)

Nyström T^1^, Schwarcz E^2^, Dahlqvist S ^3,4^, Wijkman M^5^, Ekelund M^6^, Holmer H^7^ Bolinder J^8^, Hellman J^9^, Imberg H^10,11^, Hirsch IB^12^, Lind M^3, 4, 13^

1 Department of Clinical Science and Education, Södersjukhuset, Karolinska Institutet, Stockholm, Sweden

2 Department of Internal Medicine, Faculty of Medicine and Health, Örebro University, Örebro, Sweden

3 Department of Medicine, NU-Hospital Group, Uddevalla Sweden

4 Department of Molecular and Clinical Medicine, University of Gothenburg, Gothenburg, Sweden

5 Department of Internal Medicine and Department of Medical and Health Sciences, Linköping University, Norrköping, Sweden

6 Department of Clinical Sciences, Lund University

7 Department of Internal Medicine, Centralsjukhuset, Kristianstad, Sweden

8 Department of Medicine, Karolinska University Hospital Huddinge, Karolinska Institutet, Stockholm, Sweden

9 Department of Medical Sciences, Clinical Diabetes and Metabolism, Uppsala University, Uppsala, Sweden

10 Statistiska Konsultgruppen, Gothenburg, Sweden

11 Department of Mathematical Sciences, Chalmers University of Technology and University of Gothenburg, Gothenburg, Sweden

12 School of Medicine, University of Washington, Seattle, Washington.
13 Department of Internal Medicine, Sahlgrenska University Hospital, Gothenburg, Sweden

**Table S1.** Demographics and Patient Characteristics (FAS population excluding patients with added or changed lipid lowering medication)

| **Variable** | **Sequence 1: CGM-SMBG**  **(n=69)** | **Sequence 2: SMBG-CGM**  **(n=73)** | **p-value** |
| --- | --- | --- | --- |
| **Demographics and baseline characteristics** |  |  |  |
| Age (years) | 46.5 (13.2) 46 (20–77) n=66 | 42.4 (12.4) 42.5 (19–77) n=70 | 0.07 |
| Female sex | 30 (45.5%) | 29 (41.4%) | 0.76 |
| Caucasian race | 66 (100.0%) | 69 (98.6%) | 1.00 |
| Non Hispanic/Latino | 66 (100.0%) | 70 (100.0%) | 1.00 |
| Diabetes duration at randomization (years) | 22.9 (11.8) 22.2 (3–56.6) n=66 | 20.6 (11.5) 18.7 (1.4–44.2) n=70 | 0.27 |
| Smoking |  |  |  |
| Current | 7 (10.6%) | 9 (12.9%) |  |
| Previous | 16 (24.2%) | 15 (21.4%) |  |
| Never | 43 (65.2%) | 46 (65.7%) | 0.89 |
| **Medical history** |  |  |  |
| Previous laser photocoagulation of the retina | 12 (18.2%) | 13 (18.6%) | 1.00 |
| Previous myocardial infarction | 2 (3.0%) |  | 0.47 |
| Previous stroke | 1 (1.5%) | 1 (1.4%) | 1.00 |
| Previous bypass-graft | 1 (1.5%) |  | 0.97 |
| Previous percutaneous coronary intervention | 2 (3.0%) |  | 0.47 |
| Previous amputation |  | 1 (1.4%) | 1.00 |
| Previous diabetic foot or leg ulcer | 1 (1.5%) | 5 (7.1%) | 0.24 |
| Current diabetic foot or leg ulcer |  | 3 (4.3%) | 0.27 |
| Self-estimated number of hypoglycemias per week during the last two months | 1.95 (1.49) 2 (0–7) n=63 | 2.40 (2.25) 2 (0–12) n=65 | 0.19 |
| Number of severe hypoglycemias past year | 0.106 (0.434) 0 (0–3) n=66 | 0.043 (0.268) 0 (0–2) n=69 | 0.47 |
| Number of severe hypoglycemias past 5 years | 0.621 (1.990) 0 (0–15) n=66 | 0.333 (0.721) 0 (0–4) n=69 | 0.35 |
| Lipid lowering medication at randomization | 31 (47.0%) | 32 (45.7%) | 1.00 |
| Number of blood pressure lowering medications at randomization |  |  |  |
| 0 | 37 (56.1%) | 49 (70.0%) |  |
| 1 | 17 (25.8%) | 14 (20.0%) |  |
| 2 | 6 (9.1%) | 2 (2.9%) |  |
| ≥3 | 6 (9.1%) | 5 (7.1%) | 0.13 |
| **HbA1c at Run-in and Randomization** |  |  |  |
| HbA1c at Run-in (mmol/mol) | 71.7 (9.0) 71 (58–103) n=66 | 71.6 (9.5) 69.5 (58–104) n=70 | 1.00 |
| HbA1c at Run-in (%) | 8.71 (0.82) 8.65 (7.46–11.58) n=66 | 8.71 (0.87) 8.51 (7.46–11.67) n=70 | 1.00 |
| HbA1c at Visit 4 Randomization (mmol/mol) | 69.3 (9.6) 68 (50–103) n=66 | 68.8 (9.3) 67 (55–102) n=69 | 0.78 |
| HbA1c at Visit 4 Randomization (%) | 8.49 (0.88) 8.38 (6.73–11.58) n=66 | 8.45 (0.85) 8.28 (7.19–11.49) n=69 | 0.78 |
| **Laboratory data at Randomization** |  |  |  |
| Total cholesterol (mmol/L) | 2.46 (0.74) 2.4 (1.4–5.6) n=65 | 2.52 (0.80) 2.4 (1–4.6) n=69 | 0.65 |
| Low-density lipoprotein (mmol/L) | 1.70 (0.57) 1.5 (0.9–3.5) n=66 | 1.49 (0.41) 1.4 (0.8–3.3) n=69 | 0.018 |
| Hi-density lipoprotein (mmol/L) | 0.955 (0.743) 0.75 (0.25–4.4) n=66 | 0.971 (0.548) 0.82 (0.39–3.9) n=69 | 0.88 |
| Triglycerides (mmol/L) | 4.58 (0.98) 4.55 (2.8–7.6) n=66 | 4.45 (0.93) 4.4 (2.4–6.7) n=69 | 0.43 |
| Apolipoprotein A1 (g/L) | 1.71 (0.34) 1.66 (1.1–2.8) n=66 | 1.55 (0.28) 1.53 (0.96–2.64) n=69 | 0.004 |
| Apolipoprotein B1 (g/L) | 0.863 (0.184) 0.85 (0.47–1.47) n=66 | 0.882 (0.216) 0.84 (0.5–1.63) n=69 | 0.59 |
| High-sensitivity C-reactive protein (mg/L) | 1.93 (2.54) 1.35 (0.2–17.9) n=66 | 3.59 (7.27) 1.6 (0.2–54.2) n=69 | 0.055 |
| Creatinine (µmol/L) | 73.5 (13.9) 71 (46–109) n=66 | 69.8 (12.4) 68 (37–109) n=69 | 0.11 |
| **Blood pressure at Randomization** |  |  |  |
| Systolic blood pressure (mmHg) | 125.0 (12.2) 126 (95–164) n=66 | 125.5 (19.8) 122 (80–200) n=69 | 0.88 |
| Diastolic blood pressure (mmHg) | 73.6 (9.9) 74 (52–92) n=66 | 74.4 (9.7) 74 (60–100) n=69 | 0.63 |
| **Physical activity at Run-in** |  |  |  |
| International Physical Activity Questionnaire (IPAQ) Total Score | 3595 (4529) 1760 (80–23730) n=59 | 3159 (3367) 2019 (198–13880) n=65 | 0.54 |
| International Physical Activity Questionnaire (IPAQ) Total Score, categorical |  |  |  |
| Inactive | 39 (59.1%) | 44 (62.9%) |  |
| Minimally Active | 7 (10.6%) | 4 (5.7%) |  |
| Vigorous Active | 20 (30.3%) | 22 (31.4%) | 0.87 |
| Data are presented as mean (SD), median (range) and numbers for continuous variables, as and number (percent) for categorical variables.  For comparison between groups, Fishers’ exact test (2 $\times$ lowest 1-sided p-value) was used for binary variables, Mantel-Haenszel Chi-square test for ordered categorical variables, and non-parametric permutation test for the difference in means for continuous variables.  **Abbreviations**: CGM, Continuous glucose monitoring; FAS, Full analysis set; SD, standard deviation; SMBG, Self-monitoring of blood glucose. | | | |

**Table S2.** Demographics and Patient Characteristics (FAS Population excluding patients with added or changed blood pressure lowering medication)

| **Variable** | **Sequence 1: CGM-SMBG**  **(n=69)** | **Sequence 2: SMBG-CGM**  **(n=73)** | **p-value** |
| --- | --- | --- | --- |
| **Demographics and baseline characteristics** |  |  |  |
| Age (years) | 45.2 (12.6) 45.5 (20–77) n=58 | 42.4 (12.4) 42 (19–77) n=65 | 0.22 |
| Female sex | 27 (46.6%) | 27 (41.5%) | 0.71 |
| Caucasian race | 58 (100.0%) | 64 (98.5%) | 1.00 |
| Non Hispanic/Latino | 58 (100.0%) | 65 (100.0%) | 1.00 |
| Diabetes duration at randomization (years) | 22.6 (11.1) 22.2 (3.1–47.3) n=58 | 20.0 (11.3) 17.7 (1.4–44.2) n=65 | 0.19 |
| Smoking |  |  |  |
| Current | 5 (8.6%) | 8 (12.3%) |  |
| Previous | 12 (20.7%) | 14 (21.5%) |  |
| Never | 41 (70.7%) | 43 (66.2%) | 0.50 |
| **Medical history** |  |  |  |
| Previous laser photocoagulation of the retina | 12 (20.7%) | 12 (18.5%) | 0.93 |
| Previous myocardial infarction | 0 (0.0%) | 0 (0.0%) | 1.00 |
| Previous stroke | 1 (1.7%) | 1 (1.5%) | 1.00 |
| Previous bypass-graft | 0 (0.0%) | 0 (0.0%) | 1.00 |
| Previous percutaneous coronary intervention | 0 (0.0%) | 0 (0.0%) | 1.00 |
| Previous amputation |  | 1 (1.5%) | 1.00 |
| Previous diabetic foot or leg ulcer | 1 (1.7%) | 4 (6.2%) | 0.44 |
| Current diabetic foot or leg ulcer |  | 3 (4.6%) | 0.29 |
| Self-estimated number of hypoglycemias per week during the last two months | 1.98 (1.46) 2 (0–7) n=55 | 2.30 (2.26) 2 (0–12) n=60 | 0.39 |
| Number of severe hypoglycemias past year | 0.086 (0.431) 0 (0–3) n=58 | 0.047 (0.278) 0 (0–2) n=64 | 0.73 |
| Number of severe hypoglycemias past 5 years | 0.655 (2.107) 0 (0–15) n=58 | 0.344 (0.739) 0 (0–4) n=64 | 0.36 |
| Lipid lowering medication at randomization | 26 (44.8%) | 32 (49.2%) | 0.76 |
| Number of blood pressure lowering medications at randomization |  |  |  |
| 0 | 36 (62.1%) | 47 (72.3%) |  |
| 1 | 15 (25.9%) | 12 (18.5%) |  |
| 2 | 2 (3.4%) | 1 (1.5%) |  |
| ≥3 | 5 (8.6%) | 5 (7.7%) | 0.38 |
| **HbA1c at Run-in and Randomization** |  |  |  |
| HbA1c at Run-in (mmol/mol) | 72.0 (9.1) 71 (58–103) n=58 | 71.2 (9.0) 70 (58–94) n=65 | 0.66 |
| HbA1c at Run-in (%) | 8.74 (0.83) 8.65 (7.46–11.58) n=58 | 8.67 (0.82) 8.56 (7.46–10.75) n=65 | 0.66 |
| HbA1c at Visit 4 Randomization (mmol/mol) | 69.2 (10.0) 68.5 (50–103) n=58 | 68.5 (8.6) 67 (55–96) n=64 | 0.71 |
| HbA1c at Visit 4 Randomization (%) | 8.48 (0.92) 8.42 (6.73–11.58) n=58 | 8.43 (0.79) 8.28 (7.19–10.94) n=64 | 0.71 |
| **Laboratory data at Randomization** |  |  |  |
| Total cholesterol (mmol/L) | 2.55 (0.74) 2.5 (1.4–5.6) n=57 | 2.47 (0.79) 2.3 (1–4.6) n=64 | 0.57 |
| Low-density lipoprotein (mmol/L) | 1.66 (0.56) 1.5 (0.9–3.5) n=58 | 1.51 (0.43) 1.4 (0.8–3.3) n=64 | 0.10 |
| High-density lipoprotein (mmol/L) | 0.961 (0.751) 0.735 (0.3–4.4) n=58 | 0.943 (0.533) 0.815 (0.39–3.9) n=64 | 0.87 |
| Triglycerides (mmol/L) | 4.63 (1.01) 4.55 (2.8–7.6) n=58 | 4.39 (0.93) 4.3 (2.4–6.7) n=64 | 0.18 |
| Apolipoprotein A1 (g/L) | 1.69 (0.35) 1.61 (1.08–2.8) n=58 | 1.56 (0.29) 1.53 (0.96–2.64) n=64 | 0.031 |
| Apolipoprotein B1 (g/L) | 0.892 (0.177) 0.88 (0.47–1.47) n=58 | 0.863 (0.204) 0.84 (0.5–1.34) n=64 | 0.41 |
| High-sensitivity C-reactive protein (mg/L) | 1.67 (1.51) 1.35 (0.2–8.6) n=58 | 3.60 (7.46) 1.6 (0.2–54.2) n=64 | 0.016 |
| Creatinine (µmol/L) | 72.1 (13.2) 70.5 (41–107) n=58 | 69.7 (12.7) 68 (37–109) n=64 | 0.30 |
| **Blood pressure at Randomization** |  |  |  |
| Systolic blood pressure (mmHg) | 124.6 (12.4) 125.5 (95–164) n=58 | 123.3 (17.3) 121 (80–170) n=64 | 0.64 |
| Diastolic blood pressure (mmHg) | 73.5 (9.7) 73.5 (52–92) n=58 | 73.1 (8.8) 70 (60–100) n=64 | 0.82 |
| **Physical activity at Run-in** |  |  |  |
| International Physical Activity Questionnaire (IPAQ) Total Score | 3773 (4824) 1569 (80–23730) n=50 | 2987 (3258) 1878 (198–13880) n=61 | 0.32 |
| International Physical Activity Questionnaire (IPAQ) Total Score, categorical |  |  |  |
| Inactive | 34 (59.6%) | 42 (64.6%) |  |
| Minimally Active | 5 (8.8%) | 4 (6.2%) |  |
| Vigorous Active | 18 (31.6%) | 19 (29.2%) | 0.66 |
| Data are presented as mean (SD), median (range) and numbers for continuous variables, as and number (percent) for categorical variables.  For comparison between groups, Fishers’ exact test (2 $\times$ lowest 1-sided p-value) was used for binary variables, Mantel-Haenszel Chi-square test for ordered categorical variables, and non-parametric permutation test for the difference in means for continuous variables.  **Abbreviations**: CGM, Continuous glucose monitoring; FAS, Full analysis set; SD, standard deviation; SMBG, Self-monitoring of blood glucose. | | | |

**Table S3.** Differences between treatments with respect to blood lipids, apolipoproteins and hsCRP levels in subjects with HbA1c reductions ≥5 mmol/mol during CGM compared to SMBG (FAS population excluding added or changed lipid lowering medication).

| **Variable** | **CGM**  **(DexCom G4)** | **Conventional therapy (SMBG)** | **Difference**  **CGM – SMBG** |
| --- | --- | --- | --- |
| Total cholesterol (mmol/L) | 4.49 (0.91) 4.6 (2.6-7.2) n=58 | 4.55 (0.99) 4.4 (2.8-7.7) n=58 | -0.05 (-0.28-0.18)  p=0.67 |
| Low-density lipoprotein (mmol/L) | 2.52 (0.75) 2.5 (1.0-5.0) n=58 | 2.54 (0.86) 2.5 (0.7-5.1) n=57 | -0.01 (-0.21-0.19)  p=0.91 |
| High-density lipoprotein (mmol/L) | 1.55 (0.43) 1.4 (1.0-2.6) n=58 | 1.54 (0.44) 1.5 (0.8-2.7) n=58 | 0.01 (0.32) 0.0 (-0.5-1.4) p=0.81 |
| Triglycerides (mmol/L) | 0.93 (0.41) 0.8 (0.3-2.5) n=58 | 1.10 (0.78) 0.8 (0.3-5.5) n=58 | -0.16 (0.66) -0.1 (-4.1-0.6) p=0.037 |
| Apolipoprotein A1 (g/L) | 1.57 (0.31) 1.5 (1.1-2.3) n=58 | 1.58 (0.30) 1.5 (1.1-2.4) n=58 | -0.01 (-0.06-0.05)  p=0.85 |
| Apolipoprotein B1 (g/L) | 0.85 (0.20) 0.9 (0.5-1.6) n=58 | 0.85 (0.22) 0.8 (0.4-1.5) n=58 | -0.00 (-0.05-0.05)  p=0.93 |
| High-sensitivity CRP (mg/L) | 3.92 (8.73) 1.0 (0.2-49.2) n=58 | 1.97 (2.24) 1.0 (0.2-9.6) n=58 | 1.96 (8.79) 0.0 (-3.7-48.9) p=0.14 |
| Data during CGM or SMBG are presented as mean (SD), median (range), and number of observations.  Differences between treatments (CGM–SMBG) are presented as mean difference with 95% confidence interval for normally distributed variables, and as mean (SD) and median (range) for non-normally distributed variables.  Analyses of normally distributed variables were performed using general linear models with sequence (CGM-SMBG or SMBG-CGM), patient (nested within sequence), period (1 or 2) and treatment as explanatory variables.  Analyses of non-normally distributed variables were performed using non-parametric permutation test for the difference in means.  Subjects with missing data on one of the treatment periods were excluded from the analyses.  **Abbreviations**: CGM, Continuous glucose monitoring; FAS, Full analysis set; SD, Standard deviation; SMBG, Self-monitoring of blood glucose. | | | |

**Table S4.** Differences between treatments with respect to blood lipids, apolipoproteins and hsCRP levels in subjects with HbA1c reductions ≥7 mmol/mol during CGM compared to SMBG (FAS population excluding added or changed lipid lowering medication).

| **Variable** | **CGM**  **(DexCom G4)** | **Conventional therapy (SMBG)** | **Difference**  **CGM – SMBG** |
| --- | --- | --- | --- |
| Total cholesterol (mmol/L) | 4.53 (0.89) 4.6 (2.6-7.2) n=52 | 4.59 (1.01) 4.4 (2.8-7.7) n=52 | -0.07 (-0.32-0.18)  p=0.58 |
| Low-density lipoprotein (mmol/L) | 2.54 (0.76) 2.5 (1.0-5.0) n=52 | 2.56 (0.90) 2.5 (0.7-5.1) n=51 | -0.03 (-0.25-0.20)  p=0.82 |
| High-density lipoprotein (mmol/L) | 1.56 (0.43) 1.4 (1.0-2.6) n=52 | 1.55 (0.43) 1.5 (0.8-2.7) n=52 | 0.01 (0.34) -0.0 (-0.5-1.4) p=0.87 |
| Triglycerides (mmol/L) | 0.95 (0.42) 0.8 (0.4-2.5) n=52 | 1.14 (0.81) 0.9 (0.3-5.5) n=52 | -0.19 (0.69) -0.1 (-4.1-0.6) p=0.036 |
| Apolipoprotein A1 (g/L) | 1.58 (0.31) 1.5 (1.1-2.3) n=52 | 1.59 (0.30) 1.5 (1.1-2.4) n=52 | -0.01 (-0.07-0.06)  p=0.84 |
| Apolipoprotein B1 (g/L) | 0.84 (0.21) 0.9 (0.5-1.6) n=52 | 0.86 (0.23) 0.8 (0.4-1.5) n=52 | -0.01 (-0.07-0.04)  p=0.67 |
| High-sensitivity CRP (mg/L) | 4.18 (9.18) 1.0 (0.2-49.2) n=52 | 2.06 (2.33) 1.1 (0.2-9.6) n=52 | 2.12 (9.26) 0.0 (-3.7-48.9) p=0.14 |
| Data during CGM or SMBG are presented as mean (SD), median (range), and number of observations.  Differences between treatments (CGM–SMBG) are presented as mean difference with 95% confidence interval for normally distributed variables, and as mean (SD) and median (range) for non-normally distributed variables.  Analyses of normally distributed variables were performed using general linear models with sequence (CGM-SMBG or SMBG-CGM), patient (nested within sequence), period (1 or 2) and treatment as explanatory variables.  Analyses of non-normally distributed variables were performed using non-parametric permutation test for the difference in means.  Subjects with missing data on one of the treatment periods were excluded from the analyses.  **Abbreviations**: CGM, Continuous glucose monitoring; FAS, Full analysis set; SD, Standard deviation; SMBG, Self-monitoring of blood glucose. | | | |

**Table S5.** Differences between treatments with respect to blood lipids, apolipoproteins and hsCRP levels in subjects with HbA1c reductions ≥10 mmol/mol during CGM compared to SMBG (FAS population excluding added or changed lipid lowering medication).

| **Variable** | **CGM**  **(DexCom G4)** | **Conventional therapy (SMBG)** | **Difference**  **CGM – SMBG** |
| --- | --- | --- | --- |
| Total cholesterol (mmol/L) | 4.58 (0.82) 4.8 (2.6-5.9) n=32 | 4.68 (1.04) 4.7 (2.8-7.7) n=32 | -0.08 (-0.37-0.21)  p=0.59 |
| LDL (mmol/L) | 2.51 (0.70) 2.6 (1.0-3.8) n=32 | 2.66 (0.95) 2.6 (0.7-5.1) n=31 | -0.13 (-0.39-0.13)  p=0.31 |
| HDL (mmol/L) | 1.62 (0.46) 1.5 (1.0-2.6) n=32 | 1.56 (0.41) 1.5 (1.0-2.4) n=32 | 0.06 (0.35) 0.0 (-0.4-1.4) p=0.32 |
| Triglycerides (mmol/L) | 1.00 (0.45) 0.9 (0.4-2.5) n=32 | 1.15 (0.94) 0.8 (0.3-5.5) n=32 | -0.14 (0.81) 0.0 (-4.1-0.6) p=0.38 |
| Apolipoprotein A1 (g/L) | 1.60 (0.33) 1.5 (1.1-2.3) n=32 | 1.58 (0.30) 1.5 (1.2-2.4) n=32 | 0.02 (-0.06-0.11)  p=0.55 |
| Apolipoprotein B1 (g/L) | 0.84 (0.19) 0.9 (0.5-1.3) n=32 | 0.85 (0.24) 0.9 (0.4-1.5) n=32 | -0.01 (-0.08-0.06)  p=0.67 |
| High-sensitivity CRP (mg/L) | 4.36 (9.52) 1.0 (0.2-49.2) n=32 | 2.06 (2.39) 1.0 (0.2-8.8) n=32 | 2.30 (9.63) 0.0 (-1.7-48.9) p=0.36 |
| Data during CGM or SMBG are presented as mean (SD), median (range), and number of observations.  Differences between treatments (CGM–SMBG) are presented as mean difference with 95% confidence interval for normally distributed variables, and as mean (SD) and median (range) for non-normally distributed variables.  Analyses of normally distributed variables were performed using general linear models with sequence (CGM-SMBG or SMBG-CGM), patient (nested within sequence), period (1 or 2) and treatment as explanatory variables.  Analyses of non-normally distributed variables were performed using non-parametric permutation test for the difference in means.  Subjects with missing data on one of the treatment periods were excluded from the analyses.  **Abbreviations**: CGM, Continuous glucose monitoring; FAS, Full analysis set; SD, Standard deviation; SMBG, Self-monitoring of blood glucose. | | | |

**Table S6.** Differences between treatments with respect to blood lipids, apolipoproteins and hsCRP levels in subjects with weight change ≤ 0.7 kg (50^th^ percentile) during CGM – SMBG (FAS population excluding added or changed lipid lowering medication).

| **Variable** | **CGM**  **(DexCom G4)** | **Conventional therapy (SMBG)** | **Difference**  **CGM – SMBG** |
| --- | --- | --- | --- |
| Total cholesterol (mmol/L) | 4.51 (0.83) 4.6 (2.6-6.5) n=59 | 4.50 (0.90) 4.3 (2.8-7.7) n=59 | 0.05 (-0.15-0.26)  p=0.60 |
| LDL (mmol/L) | 2.45 (0.68) 2.5 (1.0-4.0) n=59 | 2.44 (0.78) 2.4 (1.1-5.1) n=58 | 0.05 (-0.12-0.22)  p=0.59 |
| HDL (mmol/L) | 1.65 (0.47) 1.5 (1.0-3.1) n=59 | 1.64 (0.43) 1.7 (0.9-2.7) n=59 | 0.01 (0.29) 0.0 (-0.7-0.9) p=0.79 |
| Triglycerides (mmol/L) | 0.92 (0.52) 0.7 (0.3-3.2) n=59 | 1.00 (0.82) 0.8 (0.3-5.5) n=59 | -0.08 (0.65) 0.0 (-4.1-1.2) p=0.30 |
| Apolipoprotein A1 (g/L) | 1.66 (0.34) 1.6 (1.1-3.0) n=59 | 1.64 (0.28) 1.6 (1.1-2.4) n=59 | 0.01 (-0.06-0.07)  p=0.85 |
| Apolipoprotein B1 (g/L) | 0.84 (0.17) 0.9 (0.5-1.3) n=59 | 0.82 (0.20) 0.8 (0.4-1.5) n=59 | 0.02 (-0.03-0.06)  p=0.44 |
| High-sensitivity CRP (mg/L) | 3.29 (7.55) 1.2 (0.2-51.5) n=59 | 2.02 (2.35) 1.1 (0.2-12.0) n=59 | 1.27 (7.45) 0.1 (-4.2-50.8) p=0.18 |
| Data during CGM or SMBG are presented as mean (SD), median (range), and number of observations.  Differences between treatments (CGM–SMBG) are presented as mean difference with 95% confidence interval for normally distributed variables, and as mean (SD) and median (range) for non-normally distributed variables.  Analyses of normally distributed variables were performed using general linear models with sequence (CGM-SMBG or SMBG-CGM), patient (nested within sequence), period (1 or 2) and treatment as explanatory variables.  Analyses of non-normally distributed variables were performed using non-parametric permutation test for the difference in means.  Subjects with missing data on one of the treatment periods were excluded from the analyses.  **Abbreviations**: CGM, Continuous glucose monitoring; FAS, Full analysis set; SD, Standard deviation; SMBG, Self-monitoring of blood glucose. | | | |

**Table S7**. Differences between treatments with respect to blood lipids, apolipoproteins and hsCRP levels in subjects with weight reduction ≥1 kg (75^th^ percentile) during CGM compared to SMBG (FAS population excluding added or changed lipid lowering medication).

| **Variable** | **CGM**  **(DexCom G4)** | **Conventional therapy (SMBG)** | **Difference**  **CGM – SMBG** |
| --- | --- | --- | --- |
| Total cholesterol (mmol/L) | 4.49 (0.96) 4.6 (2.6-6.5) n=33 | 4.45 (0.86) 4.3 (2.8-6.5) n=33 | 0.07 (-0.18-0.31)  p=0.58 |
| LDL (mmol/L) | 2.45 (0.77) 2.6 (1.0-3.6) n=33 | 2.41 (0.82) 2.2 (1.1-4.3) n=33 | 0.07 (-0.13-0.26)  p=0.49 |
| HDL (mmol/L) | 1.65 (0.48) 1.5 (1.0-3.1) n=33 | 1.65 (0.40) 1.7 (0.9-2.7) n=33 | -0.00 (0.32) 0.0 (-0.7-0.9) p=1.00 |
| Triglycerides (mmol/L) | 0.91 (0.50) 0.7 (0.4-2.5) n=33 | 0.86 (0.38) 0.8 (0.3-1.9) n=33 | 0.05 (0.41) 0.0 (-0.9-1.2) p=0.61 |
| Apolipoprotein A1 (g/L) | 1.66 (0.38) 1.6 (1.1-3.0) n=33 | 1.63 (0.26) 1.6 (1.1-2.2) n=33 | 0.01 (-0.09-0.10)  p=0.91 |
| Apolipoprotein B1 (g/L) | 0.83 (0.17) 0.9 (0.5-1.2) n=33 | 0.81 (0.19) 0.8 (0.4-1.2) n=33 | 0.01 (-0.04-0.05)  p=0.74 |
| High-sensitivity CRP (mg/L) | 4.30 (9.88) 1.2 (0.5-51.5) n=33 | 1.93 (2.39) 1.1 (0.4-12.0) n=33 | 2.36 (9.86) 0.2 (-4.2-50.8) p=0.16 |
| Data during CGM or SMBG are presented as mean (SD), median (range), and number of observations.  Differences between treatments (CGM–SMBG) are presented as mean difference with 95% confidence interval for normally distributed variables, and as mean (SD) and median (range) for non-normally distributed variables.  Analyses of normally distributed variables were performed using general linear models with sequence (CGM-SMBG or SMBG-CGM), patient (nested within sequence), period (1 or 2) and treatment as explanatory variables.  Analyses of non-normally distributed variables were performed using non-parametric permutation test for the difference in means.  Subjects with missing data on one of the treatment periods were excluded from the analyses.  **Abbreviations**: CGM, Continuous glucose monitoring; FAS, Full analysis set; SD, Standard deviation; SMBG, Self-monitoring of blood glucose. | | | |

**Table S8**. Differences between treatments with respect to blood lipids, apolipoproteins and hsCRP levels in subjects with CGM SD reductions ≥0.47 mmol/L (50^th^ percentile) during CGM compared to SMBG (FAS population excluding added or changed lipid lowering medication).

| **Variable** | **CGM**  **(DexCom G4)** | **Conventional therapy (SMBG)** | **Difference**  **CGM – SMBG** |
| --- | --- | --- | --- |
| Total cholesterol (mmol/L) | 4.51 (0.87) 4.5 (2.6-7.2) n=61 | 4.42 (0.91) 4.2 (2.8-7.7) n=61 | 0.07 (-0.16-0.29)  p=0.55 |
| LDL (mmol/L) | 2.56 (0.75) 2.6 (1.0-5.0) n=61 | 2.48 (0.79) 2.4 (1.1-5.1) n=60 | 0.05 (-0.14-0.24)  p=0.60 |
| HDL (mmol/L) | 1.51 (0.44) 1.4 (0.8-3.1) n=61 | 1.49 (0.39) 1.4 (0.9-2.7) n=61 | 0.02 (0.31) 0.0 (-0.5-1.4) p=0.66 |
| Triglycerides (mmol/L) | 1.00 (0.52) 0.9 (0.3-3.2) n=61 | 1.06 (0.83) 0.8 (0.3-5.5) n=61 | -0.05 (0.63) 0.0 (-4.1-1.0) p=0.65 |
| Apolipoprotein A1 (g/L) | 1.56 (0.33) 1.5 (1.1-3.0) n=61 | 1.54 (0.27) 1.5 (1.0-2.4) n=61 | 0.02 (-0.05-0.08)  p=0.59 |
| Apolipoprotein B1 (g/L) | 0.86 (0.22) 0.9 (0.5-1.6) n=61 | 0.85 (0.22) 0.8 (0.4-1.5) n=61 | 0.01 (-0.04-0.07)  p=0.57 |
| High-sensitivity CRP (mg/L) | 4.65 (8.78) 1.4 (0.2-49.2) n=61 | 3.23 (6.09) 1.2 (0.2-44.5) n=61 | 1.42 (9.53) 0.0 (-30.7-48.9) p=0.21 |
| Data during CGM or SMBG are presented as mean (SD), median (range), and number of observations.  Differences between treatments (CGM–SMBG) are presented as mean difference with 95% confidence interval for normally distributed variables, and as mean (SD) and median (range) for non-normally distributed variables.  Analyses of normally distributed variables were performed using general linear models with sequence (CGM-SMBG or SMBG-CGM), patient (nested within sequence), period (1 or 2) and treatment as explanatory variables.  Analyses of non-normally distributed variables were performed using non-parametric permutation test for the difference in means.  Subjects with missing data on one of the treatment periods were excluded from the analyses.  **Abbreviations**: CGM, Continuous glucose monitoring; FAS, Full analysis set; SD, Standard deviation; SMBG, Self-monitoring of blood glucose. | | | |

**Table S9.** Differences between treatments with respect to blood lipids, apolipoproteins and hsCRP levels in subjects with CGM SD reductions ≥0.91 mmol/L (75^th^ percentile) during CGM compared to SMBG (FAS population excluding added or changed lipid lowering medication).

| **Variable** | **CGM**  **(DexCom G4)** | **Conventional therapy (SMBG)** | **Difference**  **CGM – SMBG** |
| --- | --- | --- | --- |
| Total cholesterol (mmol/L) | 4.52 (0.87) 4.6 (3.2-7.2) n=31 | 4.58 (0.96) 4.4 (3.1-7.7) n=31 | -0.12 (-0.52-0.27)  p=0.52 |
| LDL (mmol/L) | 2.54 (0.75) 2.5 (1.4-5.0) n=31 | 2.63 (0.87) 2.6 (1.3-5.1) n=30 | -0.14 (-0.48-0.20)  p=0.40 |
| HDL (mmol/L) | 1.48 (0.38) 1.5 (1.0-2.6) n=31 | 1.45 (0.32) 1.4 (0.9-2.3) n=31 | 0.03 (0.34) 0.0 (-0.5-1.4) p=0.79 |
| Triglycerides (mmol/L) | 1.11 (0.58) 1.1 (0.3-3.2) n=31 | 1.24 (1.05) 1.0 (0.4-5.5) n=31 | -0.13 (0.83) 0.0 (-4.1-0.6) p=0.54 |
| Apolipoprotein A1 (g/L) | 1.57 (0.31) 1.5 (1.1-2.3) n=31 | 1.55 (0.28) 1.5 (1.2-2.4) n=31 | 0.02 (-0.08-0.11)  p=0.71 |
| Apolipoprotein B1 (g/L) | 0.87 (0.23) 0.9 (0.5-1.6) n=31 | 0.90 (0.23) 0.9 (0.5-1.5) n=31 | -0.03 (-0.12-0.06)  p=0.51 |
| High-sensitivity CRP (mg/L) | 6.58 (11.43) 1.7 (0.2-49.2) n=31 | 2.81 (2.83) 1.4 (0.2-9.6) n=31 | 3.77 (11.76) 0.3 (-3.7-48.9) p=0.042 |
| Data during CGM or SMBG are presented as mean (SD), median (range), and number of observations.  Differences between treatments (CGM–SMBG) are presented as mean difference with 95% confidence interval for normally distributed variables, and as mean (SD) and median (range) for non-normally distributed variables.  Analyses of normally distributed variables were performed using general linear models with sequence (CGM-SMBG or SMBG-CGM), patient (nested within sequence), period (1 or 2) and treatment as explanatory variables.  Analyses of non-normally distributed variables were performed using non-parametric permutation test for the difference in means.  Subjects with missing data on one of the treatment periods were excluded from the analyses.  **Abbreviations**: CGM, Continuous glucose monitoring; FAS, Full analysis set; SD, Standard deviation; SMBG, Self-monitoring of blood glucose. | | | |

**Table S10.** Differences between treatments with respect to blood lipids, apolipoproteins and hsCRP levels in subjects with CGM CV reductions ≥3.4 percentage points (50^th^ percentile) during CGM compared to SMBG (FAS population excluding added or changed lipid lowering medication).

| **Variable** | **CGM**  **(DexCom G4)** | **Conventional therapy (SMBG)** | **Difference**  **CGM – SMBG** |
| --- | --- | --- | --- |
| Total cholesterol (mmol/L) | 4.56 (0.87) 4.5 (2.6-7.2) n=60 | 4.42 (0.76) 4.4 (2.8-6.5) n=60 | 0.13 (-0.06-0.32)  p=0.17 |
| LDL (mmol/L) | 2.56 (0.74) 2.5 (1.0-5.0) n=59 | 2.43 (0.71) 2.4 (1.1-4.4) n=60 | 0.12 (-0.04-0.28)  p=0.13 |
| HDL (mmol/L) | 1.56 (0.47) 1.5 (0.9-3.1) n=60 | 1.58 (0.42) 1.6 (0.9-2.8) n=60 | -0.01 (0.26) 0.0 (-0.5-0.9) p=0.69 |
| Triglycerides (mmol/L) | 0.99 (0.65) 0.8 (0.3-4.1) n=60 | 0.94 (0.56) 0.8 (0.3-4.0) n=60 | 0.05 (0.41) 0.0 (-0.8-2.4) p=0.39 |
| Apolipoprotein A1 (g/L) | 1.58 (0.34) 1.5 (1.1-3.0) n=60 | 1.61 (0.27) 1.6 (1.2-2.4) n=60 | -0.02 (-0.08-0.04)  p=0.47 |
| Apolipoprotein B1 (g/L) | 0.86 (0.22) 0.9 (0.5-1.6) n=60 | 0.83 (0.20) 0.8 (0.4-1.4) n=60 | 0.03 (-0.01-0.07)  p=0.14 |
| High-sensitivity CRP (mg/L) | 4.01 (7.53) 1.4 (0.2-49.2) n=60 | 3.24 (6.12) 1.4 (0.2-44.5) n=60 | 0.77 (8.28) -0.0 (-30.7-48.9) p=0.43 |
| Data during CGM or SMBG are presented as mean (SD), median (range), and number of observations.  Differences between treatments (CGM–SMBG) are presented as mean difference with 95% confidence interval for normally distributed variables, and as mean (SD) and median (range) for non-normally distributed variables.  Analyses of normally distributed variables were performed using general linear models with sequence (CGM-SMBG or SMBG-CGM), patient (nested within sequence), period (1 or 2) and treatment as explanatory variables.  Analyses of non-normally distributed variables were performed using non-parametric permutation test for the difference in means.  Subjects with missing data on one of the treatment periods were excluded from the analyses.  **Abbreviations**: CGM, Continuous glucose monitoring; FAS, Full analysis set; SD, Standard deviation; SMBG, Self-monitoring of blood glucose. | | | |

**Table S11**. Differences between treatments with respect to blood lipids, apolipoproteins and hsCRP levels in subjects with CGM CV reductions ≥8.0 percentage points (75^th^ percentile) during CGM compared to SMBG (FAS population excluding added or changed lipid lowering medication).

| **Variable** | **CGM**  **(DexCom G4)** | **Conventional therapy (SMBG)** | **Difference**  **CGM – SMBG** |
| --- | --- | --- | --- |
| Total cholesterol (mmol/L) | 4.44 (0.73) 4.5 (3.1-5.8) n=31 | 4.42 (0.77) 4.2 (3.2-6.5) n=31 | 0.02 (-0.22-0.26)  p=0.87 |
| LDL (mmol/L) | 2.37 (0.59) 2.3 (1.4-3.6) n=31 | 2.37 (0.70) 2.2 (1.4-4.3) n=31 | 0.00 (-0.18-0.18)  p=0.96 |
| HDL (mmol/L) | 1.65 (0.48) 1.6 (1.0-3.1) n=31 | 1.67 (0.38) 1.6 (0.9-2.7) n=31 | -0.02 (0.29) 0.0 (-0.5-0.9) p=0.77 |
| Triglycerides (mmol/L) | 0.93 (0.54) 0.8 (0.3-3.2) n=31 | 0.88 (0.63) 0.7 (0.4-4.0) n=31 | 0.05 (0.27) 0.1 (-0.8-0.5) p=0.31 |
| Apolipoprotein A1 (g/L) | 1.64 (0.38) 1.6 (1.1-3.0) n=31 | 1.66 (0.25) 1.6 (1.2-2.4) n=31 | -0.02 (-0.12-0.09)  p=0.75 |
| Apolipoprotein B1 (g/L) | 0.81 (0.17) 0.8 (0.5-1.3) n=31 | 0.81 (0.20) 0.8 (0.6-1.3) n=31 | 0.00 (-0.04-0.05)  p=0.87 |
| High-sensitivity CRP (mg/L) | 5.57 (9.98) 1.4 (0.2-49.2) n=31 | 4.06 (8.11) 1.4 (0.2-44.5) n=31 | 1.51 (11.51) 0.0 (-30.7-48.9) p=0.45 |
| Data during CGM or SMBG are presented as mean (SD), median (range), and number of observations.  Differences between treatments (CGM–SMBG) are presented as mean difference with 95% confidence interval for normally distributed variables, and as mean (SD) and median (range) for non-normally distributed variables.  Analyses of normally distributed variables were performed using general linear models with sequence (CGM-SMBG or SMBG-CGM), patient (nested within sequence), period (1 or 2) and treatment as explanatory variables.  Analyses of non-normally distributed variables were performed using non-parametric permutation test for the difference in means.  Subjects with missing data on one of the treatment periods were excluded from the analyses.  **Abbreviations**: CGM, Continuous glucose monitoring; FAS, Full analysis set; SD, Standard deviation; SMBG, Self-monitoring of blood glucose. | | | |

**Table S12**. Differences between treatments with respect to blood lipids, apolipoproteins and hsCRP levels in subjects with MAGE reductions ≥1.03 mmol/L (50^th^ percentile) during CGM compared to SMBG (FAS population excluding added or changed lipid lowering medication).

| **Variable** | **CGM**  **(DexCom G4)** | **Conventional therapy (SMBG)** | **Difference**  **CGM – SMBG** |
| --- | --- | --- | --- |
| Total cholesterol (mmol/L) | 4.57 (0.83) 4.5 (3.2-7.2) n=56 | 4.41 (0.89) 4.2 (2.8-7.7) n=56 | 0.11 (-0.12-0.35)  p=0.34 |
| LDL (mmol/L) | 2.58 (0.72) 2.5 (1.5-5.0) n=56 | 2.45 (0.79) 2.4 (0.7-5.1) n=56 | 0.09 (-0.11-0.30)  p=0.36 |
| HDL (mmol/L) | 1.53 (0.45) 1.4 (1.0-3.1) n=56 | 1.48 (0.34) 1.4 (0.9-2.3) n=56 | 0.05 (0.32) 0.0 (-0.4-1.4) p=0.34 |
| Triglycerides (mmol/L) | 1.02 (0.52) 0.9 (0.3-3.2) n=56 | 1.03 (0.61) 0.8 (0.4-4.0) n=56 | -0.01 (0.40) 0.0 (-1.1-1.2) p=0.69 |
| Apolipoprotein A1 (g/L) | 1.58 (0.35) 1.5 (1.1-3.0) n=56 | 1.56 (0.25) 1.5 (1.2-2.4) n=56 | 0.03 (-0.04-0.10)  p=0.40 |
| Apolipoprotein B1 (g/L) | 0.87 (0.21) 0.9 (0.5-1.6) n=56 | 0.85 (0.21) 0.8 (0.4-1.5) n=56 | 0.02 (-0.04-0.07)  p=0.57 |
| High-sensitivity CRP (mg/L) | 4.81 (9.10) 1.4 (0.2-49.2) n=56 | 3.51 (6.30) 1.4 (0.2-44.5) n=56 | 1.30 (10.04) -0.1 (-30.7-48.9) p=0.27 |
| Data during CGM or SMBG are presented as mean (SD), median (range), and number of observations.  Differences between treatments (CGM–SMBG) are presented as mean difference with 95% confidence interval for normally distributed variables, and as mean (SD) and median (range) for non-normally distributed variables.  Analyses of normally distributed variables were performed using general linear models with sequence (CGM-SMBG or SMBG-CGM), patient (nested within sequence), period (1 or 2) and treatment as explanatory variables.  Analyses of non-normally distributed variables were performed using non-parametric permutation test for the difference in means.  Subjects with missing data on one of the treatment periods were excluded from the analyses.  **Abbreviations**: CGM, Continuous glucose monitoring; FAS, Full analysis set; SD, Standard deviation; SMBG, Self-monitoring of blood glucose. | | | |

**Table S13**. Differences between treatments with respect to blood lipids, apolipoproteins and hsCRP levels in subjects with MAGE reductions ≥2.13 mmol/L (75^th^ percentile) during CGM compared to SMBG (FAS population excluding added or changed lipid lowering medication).

| **Variable** | **CGM**  **(DexCom G4)** | **Conventional therapy (SMBG)** | **Difference**  **CGM – SMBG** |
| --- | --- | --- | --- |
| Total cholesterol (mmol/L) | 4.51 (0.64) 4.6 (3.4-5.8) n=28 | 4.43 (0.74) 4.5 (3.1-6.5) n=28 | 0.07 (-0.17-0.31)  p=0.54 |
| LDL (mmol/L) | 2.42 (0.54) 2.4 (1.5-3.3) n=28 | 2.44 (0.68) 2.4 (1.3-4.3) n=28 | -0.03 (-0.24-0.18)  p=0.76 |
| HDL (mmol/L) | 1.64 (0.56) 1.5 (1.0-3.1) n=28 | 1.54 (0.36) 1.5 (0.9-2.3) n=28 | 0.10 (0.38) 0.0 (-0.4-1.4) p=0.21 |
| Triglycerides (mmol/L) | 1.04 (0.53) 0.9 (0.4-3.2) n=28 | 0.97 (0.67) 0.8 (0.6-4.0) n=28 | 0.06 (0.30) 0.1 (-0.8-0.5) p=0.30 |
| Apolipoprotein A1 (g/L) | 1.64 (0.44) 1.5 (1.1-3.0) n=28 | 1.59 (0.29) 1.5 (1.2-2.4) n=28 | 0.06 (-0.06-0.17)  p=0.35 |
| Apolipoprotein B1 (g/L) | 0.83 (0.17) 0.9 (0.5-1.3) n=28 | 0.85 (0.20) 0.8 (0.5-1.3) n=28 | -0.01 (-0.07-0.04)  p=0.59 |
| High-sensitivity CRP (mg/L) | 6.20 (10.35) 1.7 (0.2-49.2) n=28 | 4.43 (8.51) 1.0 (0.3-44.5) n=28 | 1.77 (12.09) 0.1 (-30.7-48.9) p=0.36 |
| Data during CGM or SMBG are presented as mean (SD), median (range), and number of observations.  Differences between treatments (CGM–SMBG) are presented as mean difference with 95% confidence interval for normally distributed variables, and as mean (SD) and median (range) for non-normally distributed variables.  Analyses of normally distributed variables were performed using general linear models with sequence (CGM-SMBG or SMBG-CGM), patient (nested within sequence), period (1 or 2) and treatment as explanatory variables.  Analyses of non-normally distributed variables were performed using non-parametric permutation test for the difference in means.  Subjects with missing data on one of the treatment periods were excluded from the analyses.  **Abbreviations**: CGM, Continuous glucose monitoring; FAS, Full analysis set; SD, Standard deviation; SMBG, Self-monitoring of blood glucose. | | | |

**Table S14.** Differences between treatments with respect to blood lipids, apolipoproteins and hsCRP levels in subjects with proportion time in range increase ≥3.0 percentage points (50^th^ percentile) during CGM compared to SMBG (FAS population excluding added or changed lipid lowering medication).

| **Variable** | **CGM**  **(DexCom G4)** | **Conventional therapy (SMBG)** | **Difference**  **CGM – SMBG** |
| --- | --- | --- | --- |
| Total cholesterol (mmol/L) | 4.41 (0.75) 4.5 (2.9-6.3) n=57 | 4.51 (0.92) 4.3 (2.8-7.7) n=57 | -0.13 (-0.32-0.07)  p=0.19 |
| LDL (mmol/L) | 2.44 (0.62) 2.4 (1.2-4.0) n=57 | 2.50 (0.79) 2.4 (0.7-5.1) n=57 | -0.08 (-0.25-0.08)  p=0.32 |
| HDL (mmol/L) | 1.53 (0.43) 1.4 (1.0-2.6) n=57 | 1.55 (0.41) 1.4 (0.9-2.7) n=57 | -0.02 (0.31) 0.0 (-0.7-1.4) p=0.65 |
| Triglycerides (mmol/L) | 0.97 (0.49) 0.8 (0.4-3.2) n=57 | 1.01 (0.62) 0.8 (0.4-4.0) n=57 | -0.04 (0.38) 0.0 (-1.1-0.6) p=0.41 |
| Apolipoprotein A1 (g/L) | 1.57 (0.28) 1.5 (1.1-2.3) n=57 | 1.58 (0.28) 1.5 (1.2-2.4) n=57 | -0.01 (-0.07-0.05)  p=0.81 |
| Apolipoprotein B1 (g/L) | 0.82 (0.18) 0.8 (0.5-1.3) n=57 | 0.84 (0.21) 0.8 (0.4-1.5) n=57 | -0.02 (-0.06-0.03)  p=0.44 |
| High-sensitivity CRP (mg/L) | 4.16 (8.99) 1.4 (0.2-51.5) n=57 | 2.71 (5.97) 1.3 (0.2-44.5) n=57 | 1.44 (10.03) 0.0 (-30.7-50.8) p=0.34 |
| Data during CGM or SMBG are presented as mean (SD), median (range), and number of observations.  Differences between treatments (CGM–SMBG) are presented as mean difference with 95% confidence interval for normally distributed variables, and as mean (SD) and median (range) for non-normally distributed variables.  Analyses of normally distributed variables were performed using general linear models with sequence (CGM-SMBG or SMBG-CGM), patient (nested within sequence), period (1 or 2) and treatment as explanatory variables.  Analyses of non-normally distributed variables were performed using non-parametric permutation test for the difference in means.  Subjects with missing data on one of the treatment periods were excluded from the analyses.  **Abbreviations**: CGM, Continuous glucose monitoring; FAS, Full analysis set; SD, Standard deviation; SMBG, Self-monitoring of blood glucose. | | | |

**Table S15.** Differences between treatments with respect to blood lipids, apolipoproteins and hsCRP levels in subjects with proportion time in range increase ≥11.1 percentage points (75^th^ percentile) during CGM compared to SMBG (FAS population excluding added or changed lipid lowering medication).

| **Variable** | **CGM**  **(DexCom G4)** | **Conventional therapy (SMBG)** | **Difference**  **CGM – SMBG** |
| --- | --- | --- | --- |
| Total cholesterol (mmol/L) | 4.41 (0.70) 4.6 (3.0-5.9) n=28 | 4.52 (0.91) 4.3 (2.8-7.7) n=28 | -0.20 (-0.55-0.15)  p=0.26 |
| LDL (mmol/L) | 2.50 (0.63) 2.6 (1.4-3.5) n=28 | 2.54 (0.82) 2.6 (0.7-5.1) n=28 | -0.14 (-0.43-0.15)  p=0.33 |
| HDL (mmol/L) | 1.46 (0.34) 1.4 (1.0-2.3) n=28 | 1.52 (0.38) 1.4 (1.0-2.3) n=28 | -0.06 (0.22) 0.0 (-0.7-0.3) p=0.38 |
| Triglycerides (mmol/L) | 1.01 (0.44) 0.9 (0.5-2.5) n=28 | 1.01 (0.50) 0.8 (0.4-2.1) n=28 | -0.00 (0.42) 0.1 (-1.1-0.6) p=0.93 |
| Apolipoprotein A1 (g/L) | 1.51 (0.25) 1.5 (1.1-2.2) n=28 | 1.54 (0.31) 1.5 (1.2-2.4) n=28 | -0.01 (-0.10-0.08)  p=0.84 |
| Apolipoprotein B1 (g/L) | 0.82 (0.17) 0.8 (0.5-1.2) n=28 | 0.83 (0.20) 0.8 (0.4-1.5) n=28 | -0.02 (-0.09-0.06)  p=0.60 |
| High-sensitivity CRP (mg/L) | 5.02 (11.76) 1.3 (0.2-51.5) n=28 | 1.92 (2.28) 1.1 (0.2-8.8) n=28 | 3.10 (11.93) -0.1 (-1.7-50.8) p=0.44 |
| Data during CGM or SMBG are presented as mean (SD), median (range), and number of observations.  Differences between treatments (CGM–SMBG) are presented as mean difference with 95% confidence interval for normally distributed variables, and as mean (SD) and median (range) for non-normally distributed variables.  Analyses of normally distributed variables were performed using general linear models with sequence (CGM-SMBG or SMBG-CGM), patient (nested within sequence), period (1 or 2) and treatment as explanatory variables.  Analyses of non-normally distributed variables were performed using non-parametric permutation test for the difference in means.  Subjects with missing data on one of the treatment periods were excluded from the analyses.  **Abbreviations**: CGM, Continuous glucose monitoring; FAS, Full analysis set; SD, Standard deviation; SMBG, Self-monitoring of blood glucose. | | | |

Figure S1


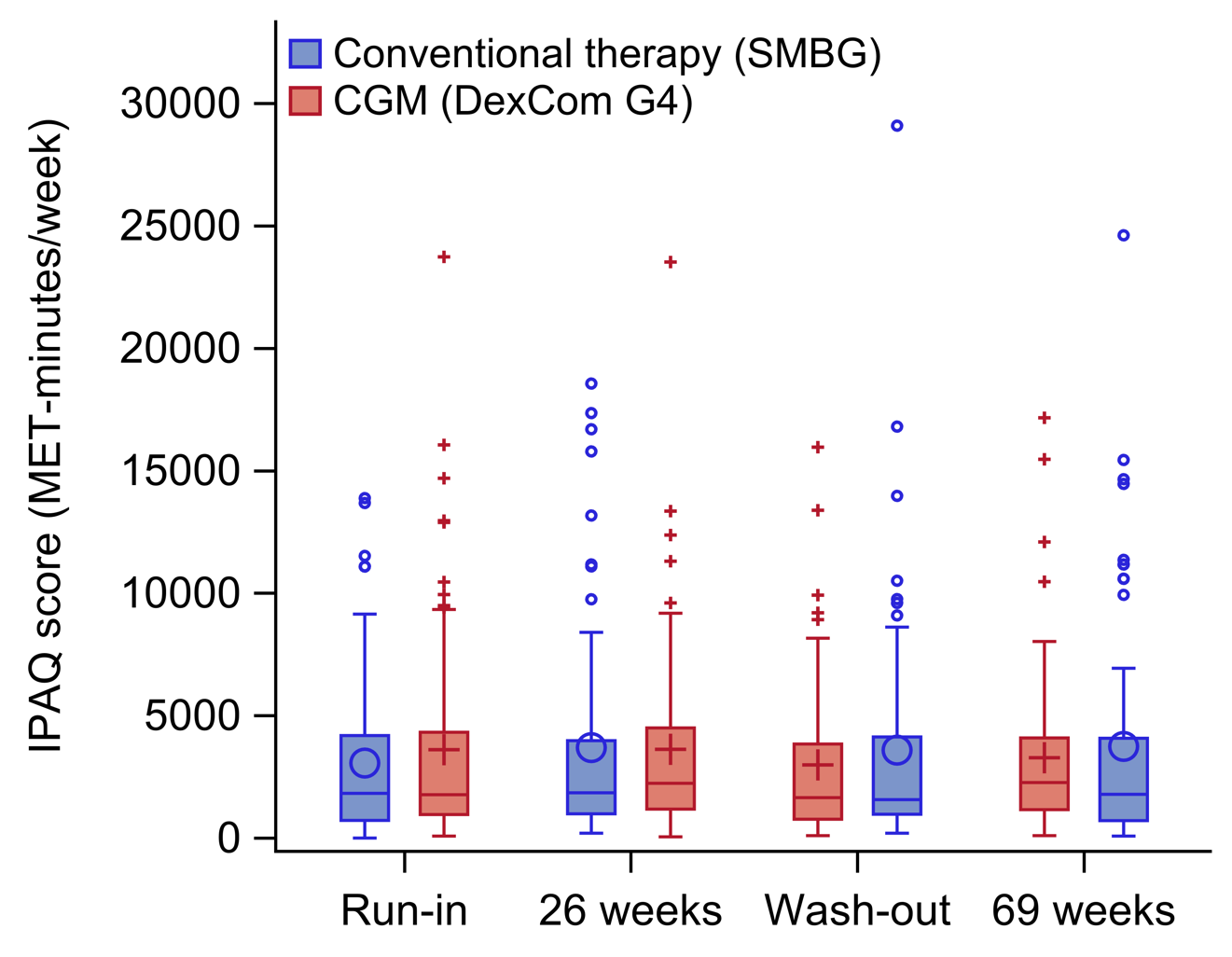

Supplement: sj-docx-1-dst-10.1177_19322968221101916 – Supplemental material for Evaluation of Effects of Continuous Glucose Monitoring on Physical Activity Habits and Blood Lipid Levels in Persons With Type 1 Diabetes Managed With MDI: An Analysis Based on the GOLD Randomized Trial (GOLD 8) [file sj-docx-1-dst-10.1177_19322968221101916.docx]
